# Nonthermal Ultrafast Optical Control of Magnetization Dynamics by Linearly Polarized Light in Metallic Ferromagnet

**DOI:** 10.1002/advs.202205903

**Published:** 2023-01-03

**Authors:** Jingyu Shi, Zirui Zhao, Yu Dai, Jiang He, Tao Li, En Liang, Jun Wang, Gang Ni, Chuanxiang Sheng, Di Wu, Shiming Zhou, Liangyao Chen, Haibin Zhao

**Affiliations:** ^1^ Key Laboratory of Micro & Nano Photonic Structures (MOE) and Shanghai Ultra‐precision Optical Manufacturing Engineering Research Center Department of Optical Science and Engineering Fudan University Shanghai 200433 China; ^2^ Basic Experimental Teaching Center Shaanxi Normal University Xi'an 710062 China; ^3^ School of Physics Science and Engineering Tongji University Shanghai 200092 China; ^4^ National Laboratory of Solid State Microstructures Department of Physics Nanjing University Nanjing 210093 China; ^5^ Shanghai Frontiers Science Research Base of Intelligent Optoelectronics and Perception Institute of Optoelectronics Fudan University Shanghai 200433 China

**Keywords:** ferroelectric polarization, magnetization precession, metallic ferromagnet, optical rectification effect, ultrafast nonthermal effect

## Abstract

Coherent optical control of the magnetization in ferromagnetic (FM) mediums using ultrafast nonthermal effect paves a promising avenue to improve the speed and repetition rate of the magnetization manipulation. Whereas previously, only heat‐induced or helicity‐dependent magnetization dynamics are demonstrated in metallic ferromagnets. Here, the linearly‐polarized light control of magnetization is demonstrated in FM Co coupled with ferroelectric (FE) BiFeO_3_ by tuning the light polarization direction. It is revealed that in the Co/BiFeO_3_ heterostructure excited by femtosecond laser pulses, the magnetization precession amplitude follows a sinusoidal dependence on the laser polarization direction. This nonthermal control of coherent magnetization rotation is attributed to the optical rectification effect in the BiFeO_3_ layer, which yields a FE polarization depending on the light polarization, and the subsequent modulation of magnetic energy in Co by the electrostriction‐induced strain. This work demonstrates an effective route to nonthermally manipulate the ultrafast magnetization dynamics in metallic ferromagnets.

## Graphical abstract

A new strategy of ultrafast nonthermal control of magnetization is adopted by coupling the metallic ferromagnet with the ferroelectric material. It utilizes the advantages of pronounced optical rectification effect and ferroelastic response in BiFeO_3_. The laser irradiation on the Co/BiFeO_3_ heterostructure leads to a sinusoidal dependence of ultrafast magnetic torque impinged upon the magnetization precession on the light polarization direction.

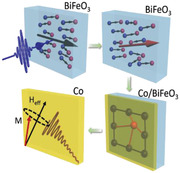

## 1 Introduction

Nonthermal optical stimulation of coherent magnetization dynamics has great value for fast‐speed information storage devices employing the magnetic medium owing to its negligible heat accumulation and high repetition rate.^[^ ^1^, ^2^, ^3^, ^4^ ^]^ In recent years, femtosecond laser pulse‐induced non‐thermal stimulation of magnetization rotation and switching has been demonstrated, offering great prospects to improve the efficiency and speed of the magnetization manipulation.^[^ ^5^, ^6^, ^7^, ^8^, ^9^, ^10^, ^11^, ^12^, ^13^, ^14^, ^15^, ^16^ ^]^ The magnetization dynamics induced by the non‐thermal optical effect usually show a strong dependence on the pump light polarization.

The circularly‐polarized light excitation can cause the inverse Faraday effect related to the impulsive stimulated Raman magnetic scattering independent of photon absorption,^[^ ^6^, ^7^ ^]^ and it can also induce the optical spin transfer torque as a result of the injection of spin‐polarized carriers in the ferromagnetic (FM) semiconductor involving the photon absorption.^[^ ^8^, ^9^ ^]^ For these effects, the left‐handed and right‐handed circular‐polarized light pulses trigger transient magnetic moments or spin‐polarized electrons with opposite orientations.^[^ ^10^ ^]^ The non‐thermal excitation mechanisms correlated with the linearly polarized light interactions include the photoinduced magnetic anisotropy effect^[^ ^11^, ^12^, ^13^ ^]^ and the inverse Cotton–Mouton effect,^[^ ^14^, ^15^, ^16^ ^]^ which exist mainly in the insulating magnetic oxides where the optically stimulated magnetization dynamics changes its phase or magnitude upon tuning the pump polarization direction.^[^ ^17^, ^18^, ^19^ ^]^

Although the circular‐polarized femtosecond laser pulses can be used to nonthermally excite and coherently control the spin dynamics in FM metals via the inverse Faraday effect,^[^ ^20^, ^21^, ^22^ ^]^ it is remained to be demonstrated how to coherently control magnetization in the FM metals using the linearly polarized light. To this end, we propose to adopt a strategy of ultrafast nonthermal control of magnetization in FM metal Co coupled with the ferroelectric (FE) material BiFeO_3_ by taking into account the advantages of pronounced optical rectification effect (ORE) and ferroelastic response in BiFeO_3_. When the FE is under intense illumination, the second‐order optical response combined with a linear term is well known to induce an extra FE polarization, and this FE polarization can consequently generate electrostriction in the system to modulate the magnetic energy of Co, depending on the light polarization direction.

Actually, the photovoltaic response, induced by the ORE, depending on the light polarization direction was already observed in bulk rhombohedral FE BiFeO_3_ crystals.^[^ ^23^ ^]^ It was also found that the BiFeO_3_ crystal exhibits a pronounced photostriction effect under linearly polarized light illumination, and its elongation critically relies on the polarization direction.^[^ ^24^ ^]^ In this case, the photostriction effect can be understood as the superposition of photovoltaic and converse piezoelectric effects. Recently, it was further demonstrated that the strain characteristics of the FE BiFeO_3_ impinged by the photostriction effect can modulate magnetic anisotropy of the adjacent FM Ni layer in a Ni/BiFeO_3_ heterostructure.^[^ ^25^ ^]^ This result confirms the scientific feasibility of the FM‐FE coupling system using the optical modulation scenario. However, until now, the magnetic dynamics response in FM‐FE systems by the light‐induced photostriction effect has been elusive.

In this paper, we have demonstrated the ultrafast nonthermal control of the magnetization precession dynamics in Co/BiFeO_3_ by using linearly polarized femtosecond laser excitation. We utilized the time‐resolved magneto‐optical Kerr effect (TRMOKE) to monitor the excited spin precession in the FM Co layer coupled with the FE BiFeO_3_ for different pump laser polarizations. It was found that the amplitude of magnetization precession with the linear polarization (*β*_E_) of pump laser pulse follows a sinusoidal profile with a periodicity of 180^o^. In contrast, the magnetization precession amplitude in the pure Co film has no such angular dependence. We also measured the ultrafast laser‐induced dynamics of the single BiFeO_3_ film and thereby obtained its light‐induced transient FE polarization. Actually, these FE polarization‐related signals also appear in the Co/BiFeO_3_ bilayer, and they both display a sinusoidal dependence on *β*_E_, which is in agreement with the deduction from the ORE. We thus believe that the nonthermal optical control of the magnetization precession in Co/BiFeO_3_ originates from the FE layer BiFeO_3_ due to its photostriction effect.

## 2 Results and Discussion

### 2.1 Illustration of the Light Induced Magnetization Rotation

**Figure 1:**
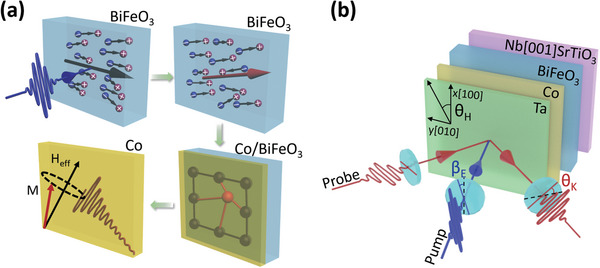
Illustration of light‐induced magnetization rotation and experimental setup. a) Illustration of the ORE‐induced FE polarization, the resultant strain due to the electrostriction effect, and the magnetization precession triggered by the strain‐induced magnetic anisotropy modulation in Co/BiFeO_3_. b) Schematic experimental setup and sample configuration, where *β*_E_ denotes the laser field angle with respect to the vertical *x* axis parallel to the [100] axis of BiFeO_3_, and *θ*_H_ represents the orientation angle of the magnetic field with the *x* axis.

As illustrated in **Figure** **1**a, upon the laser irradiation on the Co/BiFeO_3_ heterostructure, the ORE in BiFeO_3_ first leads to the direct current (dc) polarization that depends on both the polarization of pump light and its spontaneous FE polarization, and the subsequent electrostriction effect causes the strain in the bilayer. This strain modulates the magnetic anisotropy in the Co layer, resulting in the various modulation of its magnetization precession for the different light polarization. Such scenario describes a linear polarization‐dependent magnetization precession excitation in FM metals, which may represent a promising route for ultrafast nonthermal control of magnetization in conventional FM materials.

### 2.2 Sample Characterization and Experimental Configuration

The Co/BiFeO_3_ bilayer used in this study was grown on (001) oriented Nb‐doped single crystal SrTiO_3_ (STO) substrate (See Experimental Section for details). From the XRD measurements, the BiFeO_3_ has a tetragonal‐like single crystalline structure (see Figure S1, Supporting Information). Magnetization dynamics driven by ultrafast laser excitation were measured at room temperature using the pump‐probe TRMOKE system as shown in Figure 1b. The pump laser pulses of 400 nm are perpendicularly incident on the sample with the tuned polarization angle (*β*_E_). The probe laser pulses of 800 nm with *p*‐polarization are incident at a 45° oblique angle and undergo the transient polarization rotation (*θ*_K_) upon reflecting from the sample. A vector magnetic field is within the sample surface along a certain direction (*θ*_H_). The sample surface is placed in the vertical *x‐*‐*y* plane and the surface normal parallel along [001] is defined as the *z* axis. The TRMOKE system was also used to measure the *θ*_K_ related to the pump pulse induced transient FE polarization and the resultant strain in the Co/BiFeO_3_ bilayer and the pure BiFeO_3_ film. Detailed TRMOKE experimental information can be found in the Experimental Section.

### 2.3 Field Dependence of Laser Induced Magnetization Precession

**Figure 2:**
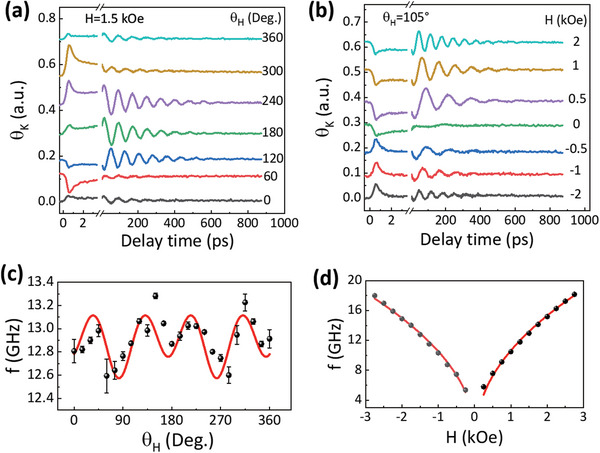
Field dependence of magnetization precession. a) *θ*_K_(t) in Co/BiFeO_3_ at H = 1.5 kOe for different *θ*_H_. b) *θ*_K_(t) in Co/BiFeO_3_ at *θ*_H_ = 105° for different field strength. c) *θ*_H_ dependence of the precession frequency at H = 1.5 kOe obtained from (a). d) The field strength dependence of precession frequency at *θ*_H_ = 105° obtained from (b). The red curves in (c) and (d) denote the calculated frequencies, respectively.

Before performing the TRMOKE measurements of pump polarization dependence in Co/BiFeO_3_, we measured the magnetization dynamics excited by an *s*‐polarized pump laser for different magnetic field orientations (*θ*_H_) and strength to obtain the field impact on the magnetization precession excitation. From **Figure** **2**a, it is noted that the oscillating *θ*_K_ signals, corresponding to the uniform spin precessions excited in Co, strongly depend on *θ*_H_. The precession amplitudes display a large variation with *θ*_H_, and the precession phases switch 180° for varying *θ*_H_. With increasing field strength, the precession amplitudes first increase and then decrease, whereas the precession phases keep unchanged, even for opposite *θ*_H_ (Figure 2b). These features fully comply with the mechanism of precession excitation due to the laser‐induced modulation of in‐plane magnetic anisotropy.^[^ ^26^, ^27^ ^]^ In Figure 2a,b, we also note that the transient *θ*_K_ within the delay time *t* = 0.5 ps shows opposite signs for reversing fields. This sign reversal of *θ*_K_ signals is contributed by the ultrafast demagnetization of the in‐plane longitudinal component.^[^ ^28^, ^29^ ^]^

Due to the superposition of the magnetic field‐independent *θ*_K_ signals caused by the electric polarization discussed later, the signals within 0.5 ps have different shapes for opposite field directions. Moreover, the magnetization precession signals at the longer time scale have contributions from both oscillating longitudinal and polar magnetization components. The polar component has the same phase for the opposite magnetic fields, whereas the longitudinal one reverses its phase, and thus we can see that the precession amplitudes are also different for opposite magnetic fields, though the frequencies keep almost the same.^[^ ^26^ ^]^

The measured precession frequency with *θ*_H_ for H = 1.5 kOe, shown in Figure 2c, displays an anisotropic behavior, which also points to the in‐plane magnetic anisotropy in the Co layer. We can roughly describe this anisotropic dependence using a four‐fold magnetic anisotropy superimposed with a two‐fold one with the common hard axis along *θ*_H_ = 90^o^. The red solid curves in Figure 2c,d denote the calculated frequencies versus field orientation and strength using the formula derived from the Landau—Lifshitz–Gilbert (LLG) equation:^[^ ^30^, ^31^ ^]^

(1) $$2\pi f\;=\;\gamma \sqrt{\left[\mathrm{Hcos}\left(\theta_{H}-\theta_{M}\right)+H^{\alpha }\right]\left[\mathrm{Hcos}\left(\theta_{H}-\theta_{M}\right)+H^{\beta }\right]}$$

where *H* ^*α*^ = *H*_4_cos (4*θ*_M_) + *H*_2_cos(2*θ*_M_) and *H* ^*β*^ = 4*πM_s_* + *H*_4_[2 − cos^2^(2*θ*_M_)]/2 − *H*_2_sin^2^(*θ*_M_), and *γ* is the gyromagnetic ratio (1.8 × 10^7^ Hz/Oe for Co), *θ*_M_ denotes the orientation angle of the magnetization in the Co layer with respect to the *x* axis. *M*_S_ represents the saturated magnetization, and *H*_2_ = 20 Oe and *H*_4_ = 40 Oe denotes the two and four‐fold anisotropy fields, respectively.

The cubic magnetic easy axis along *θ*_H_ = 45^o^ actually coincides with the direction of the external field applied along the [110] of the BiFeO_3_ layer during the sputtering of the Co layer. This indicates the importance of the in situ magnetic field during the growth for forming the cubic magnetic anisotropy in the Co layer, although it is not in a perfect single crystalline structure because of the lattice mismatch between the two layers. The emergence of the uniaxial magnetic anisotropy is likely due to the presence of a net spontaneous FE polarization in BiFeO_3_ along the y//[010] direction. This FE polarization component yields a strain along the [010] axis to make it as the uniaxial hard axis. Such in‐plane FE polarization was actually observed in BiFeO_3_ films with tetragonal‐like structures.^[^ ^32^, ^33^, ^34^, ^35^ ^]^

### 2.4 Pump Polarization Dependence of Magnetization Precession

**Figure 3:**
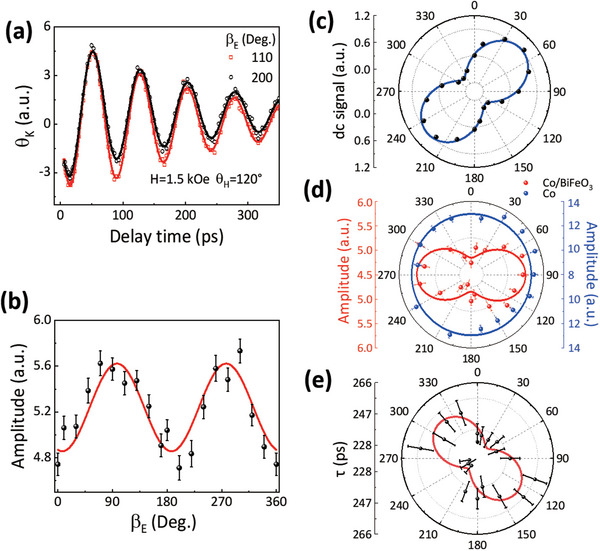
Pump polarization dependence of magnetization precession. a) The measured magnetization precession signals (open dots) in Co/BiFeO_3_ for *β*_E_=110° and 200°. The red curves are fitting results using Equation 2. b,c) *β*_E_ dependence of the precession amplitude and long‐lived dc signals (solid dots) obtained from (a). The red and blue solid curves represent fitting results using the sinusoidal functions. d) The comparison of *β*_E_ dependence of precession amplitudes in Co/BiFeO_3_ and Co. The solid dots denote the measured results. The blue curve is a circle for the eye guide. e) *β*_E_ dependence of the relaxation time *τ* (solid dots) obtained from (a). The red solid curves represent fitting results using the sinusoidal function.

The linear pump polarization (*β*_E_) dependent measurements were then performed at *θ*_H_ = 120^o^ and H = 1.5 kOe. **Figure** **3**a shows the oscillating signals corresponding to the magnetization precessions for two different *β*_E_, from which we can see an apparent difference in precession amplitude. Here, we should emphasize the constant pump fluence when rotating the half‐wave plate to tune *β*_E_. To get a deep insight into the relationship of the amplitude with *β*_E_, we fitted each *θ*_K_(t) curve with the function of

(2) $$\theta_{K}\left(t\right)\;=\;A\cdot \exp\left(-t/\tau \right)\sin\left(2\pi \mathrm{tf}+\varphi \right)+B$$

where *A*, *f*, *φ*, and *τ* represent the amplitude, frequency, initial phase, and lifetime of the precession, respectively. *B* denotes the long‐lived dc signal.

Figure 3b shows the precession amplitude (A), determined from the above fitting, with respect to the linear polarization angle *β*_E_ in the range of 360^o^. It clearly displays a sinusoidal oscillation with a period of 180^o^. The amplitude extrema appears for *s* or *p*‐polarized pump light. In addition, we note that the long‐lived dc signal (*B*) also has a sinusoidal variation with the same period of 180^o^, but it exhibits a phase difference of 45° compared to the precession amplitude variation (Figure 3c). To confirm this sinusoidal amplitude is unique for Co/BiFeO_3_, we measured a control sample of the pure Co film directly grown on the STO substrate. Its precession amplitude with *β*_E_ is isotropic, as shown in Figure 3d, and no anisotropic long‐lived dc signal is observed. We also measured the magnetization dynamics in Co/BiFeO_3_ excited by the left and right circularly polarized laser pulses. The precession amplitude for the opposite helicity pumping is nearly identical (see Figure S2, Supporting Information). Thus, we can conclude that the contribution of the inverse Faraday effect to the magnetization precession excitation is very small. Figure 3e shows the precession relaxation time *τ* as a function of *β*_E_. Despite the limited resolution, it displays an anisotropic behavior with its overall shape and phase similar to that of the *β*_E_‐dependence of the transient FE polarization signals shown below.

### 2.5 Pump Polarization Dependence of FE Polarization

**Figure 4:**
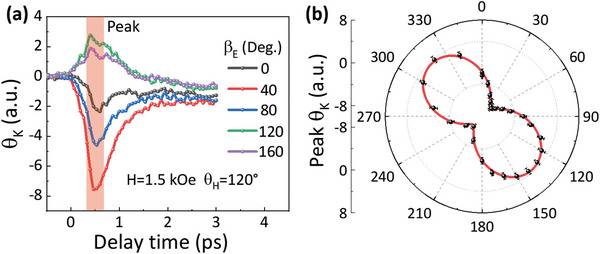
Pump polarization dependence of FE polarization in Co/BiFeO_3_. a) *θ*_K_(t) in Co/BiFeO_3_ for *t*≤3 ps and different *β*_E_. b) *β*_E_ dependence of peak *θ*_K_ values at *t* = 0.5 ps obtained from (A) (sild dots). The solid curve is the fitting result using the sinusoidal function.

To unravel the origin of anisotropic excitation of magnetization precession, we turn to investigate the *θ*_K_ signals immediately after laser excitation for different *β*_E_. Such signals can reflect the ORE‐induced FE polarization in the BiFeO_3_ layer, because the FE polarization may break the symmetry of the crystal and cause the birefringence to modulate *θ*_K_. As shown in **Figure** **4**a, *θ*_K_ rapidly rises after the pump excitation, reaches the peak at ≈0.5 ps, and attenuates greatly in 3 ps. Notably, *θ*_K_ changes its sign with varying *β*_E_. In order to clarify its variation with *β*_E_, the *θ*_K_ peak value is extracted and shown in Figure 4b, which unambiguously presents a sinusoidal dependence with the period of 180^o^, similar to the variation of the precession amplitude with *β*_E_. As discussed in the next section, the variation of this fast‐rising *θ*_K_ signal with respect to *β*_E_ is in line with the ORE induced FE polarization. Also, the transient FE polarization was proved to induce strain with a similar sinusoidal dependence on *β*_E_.^[^ ^36^ ^]^ We thus infer that the sinusoidal modulation of precession amplitude with *β*_E_ is originated from the strain modulation by the transient FE polarization. However, it should be noted that the phase of this peak *θ*_K_ oscillation related to the ORE is shifted by −45° from that of the precession amplitude, which is explained in the discussion section.

**Figure 5:**
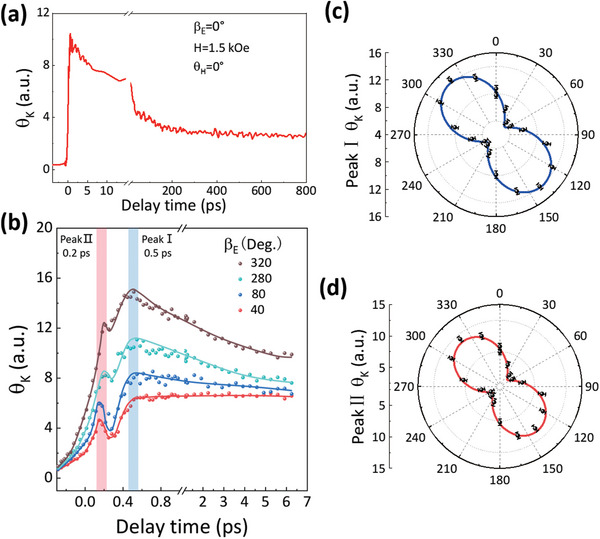
Pump polarization dependence of FE polarization in pure BiFeO_3_. a) *θ*_K_(t) in BiFeO_3_ for *t*≤800 ps. b) *θ*_K_(t) in BiFeO_3_ for *t*≤6.5 ps for different *β*_E_. The solid curves denote a guide for the eye. c,d) *β*_E_ dependence of peak I *θ*_K_ and peak II *θ*_K_. The solid curves are fitting results using the sinusoidal functions.

To confirm the above *β*_E_ dependent *θ*_K_ signals within 1 ps really arise from the ORE‐induced FE polarization in the BiFeO_3_ layer, we measured the ultrafast dynamics of a pure BiFeO_3_ film (88 nm) on Nb(001)SrTiO_3_ for different *β*_E_. Roughly, the *θ*_K_ signals show a fast rising within 1 ps and then a fast decay at the time scale of 10 ps followed by a very slow decay of several hundred ps (**Figure** **5**a). As expected, the quickly rising large *θ*_K_ signals rely strongly on *β*_E_, as shown in Figure 5b. The *θ*_K_ reaches its maximum value at ≈0.5 ps and this peak value displays a pronounced sinusoidal dependence on *β*_E_ with the period of 180^o^ (Figure 5c), in fully consistent with that observed in Co/BiFeO_3_. In contrast, the transient reflectivity does not show such anisotropic dependence on *β*_E_ (see Figure S3, Supporting Information)

In addition to this peak (peak I) at ≈0.5 ps, we note in the pure BiFeO_3_ film another small peak (peak II) at ≈0.2 ps (Figure 5b), which presents the similar *β*_E_‐dependence as peak I behaves (Figure 5d). The peak II signal actually originates from the optical Kerr effect,^[^ ^37^, ^38^ ^]^ which emerges within the laser pulse interaction duration and results in *θ*_K_ with the same variation profile as that of the ORE in our case (see Supporting Information). These small peak II signals are not present in Co/BiFeO_3_ is possibly due to the much weaker third‐order nonlinear optical response, because of the attenuation of light by the Co layer and the obscuration by the ultrafast demagnetization signals.

An intensive laser pulse propagating in the FE BiFeO_3_ can stimulate a new macroscopic electric polarization described as follows:^[^ ^36^, ^39^ ^]^

(3) $$\begin{array}{ccc} P^{\left(2\right)}\left(t\right) & = & \varepsilon_{0}\chi^{\left(3\right)}E_{\omega }\left(t\right)E_{\omega }^{*}\left(t\right)P+\varepsilon_{0}\chi^{\left(2\right)}E_{\omega }\left(t\right)E_{\omega }^{*}\left(t\right)\\ & = & P_{0}^{\left(2\right)}\left(t\right)+P_{2\omega }^{\left(2\right)}\left(t\right) \end{array}$$

where *ε*_0_ is the vacuum dielectric constant, *χ* ^(3)^ and *χ* ^(2)^ are the fourth‐ and third‐ susceptibility tensors of BiFeO_3_, respectively, *E*_*ω*_(*t*) and $E_{\omega }^{*}\left(t\right)$ denote the laser fields, *P* denotes the spontaneous FE polarization, and $P_{0}^{\left(2\right)}$ represents the direct current term of light‐induced polarization, in accordance with the ORE, while $P_{2\omega }^{\left(2\right)}\left(t\right)$ represents the contribution to the second harmonic generation.

According to the tetragonal crystal symmetry of BiFeO_3_ and the coordinate system defined in the TRMOKE experiments (Figure 1), we can derive the nonzero susceptibility components and the corresponding $P_{0}^{\left(2\right)}$ due to the ORE. In the case of an in‐plane spontaneous polarization vector *P*_y_, $P_{0}^{\left(2\right)}$ is expressed as:

(4) $$\begin{array}{ccc} P_{0y}^{2}\left(t\right) & = & \varepsilon_{0}\chi_{\mathrm{yyyy}}E_{y}E_{y}^{*}P_{y}+\varepsilon_{0}\chi_{\mathrm{yxxy}}E_{x}E_{x}^{*}P_{y}\\ & = & \varepsilon_{0}E^{2}P_{y}\left(\frac{\chi_{1}+\chi_{2}}{2}+\frac{\chi_{1}-\chi_{2}}{2}\cos2\beta_{E}\right) \end{array}$$

(5) $$P_{0x}^{2}\left(t\right)=\varepsilon_{0}\chi_{\mathrm{xxyy}}E_{x}E_{y}^{*}P_{y}+\varepsilon_{0}\chi_{\mathrm{xyxy}}E_{y}E_{x}^{*}P_{y}=\varepsilon_{0}E^{2}P_{y}\chi_{3}\sin2\beta_{E}$$

(6) $$P_{0z}^{2}\left(t\right)\;=\;0$$

Here, an in‐plane transient polarization is emergent, depending on *β*_E_ in the form of cos2*β*_E_ or sin2*β*_E_. A similar result can be obtained for the case of *P*_x_. Whereas for the spontaneous polarization vector *P*_z_, the in‐plane $P_{0}^{\left(2\right)}$ component is zero and the out‐of‐plane $P_{0}^{\left(2\right)}$ has no dependence of *β*_E_.

The above calculations show that the ORE‐induced FE polarization has a sinusoidal dependence on *β*_E_ with the period of 180^o^ in the presence of an in‐plane spontaneous FE polarization, in line with the corresponding *θ*_K_ peak values shown in Figures 4b 5c. When the total in‐plane FE polarization direction deviates from the y//[010] axis, a birefringence effect is induced for *s*‐polarized probe light, yielding the *θ*_K_ signals. From **Equations** **4** and **5**, it can be inferred that the largest deviation angle occurs for *β*_E_=45^o^ and 135^o^. Such *β*_E_ values thus should correspond to the largest modulation of the probe light polarization, which actually explains the observed *θ*_K_ extrema at *β*_E_=45^o^ and 135^o^. For the pure BiFeO_3_ film, the *θ*_K_ signals are all positive is likely due to the superposition of the contribution from the modulated spontaneous polarization independent of *β*_E_. Whereas for the Co/BiFeO_3_ bilayer, the ultrafast demagnetization signal from Co is further superimposed, thus affecting the sign of *θ*_K_ signals.

As described at the beginning of the results section, in the presence of light‐induced FE polarization, the electrostriction effect in the BiFeO_3_ causes the strain in the bilayer system. This strain modulates the magnetic anisotropy in the Co layer and provides an extra magnetic torque, in addition to the demagnetization related torque as a result of the heating effect, to drive the magnetization rotation followed by the periodic precession after the torque is nearly vanished. However, in contrast to the FE polarization induced *θ*_K_, the largest precession amplitude must appear when the magnetic energy difference between the local easy axis along 135^o^ and the hard axis along 90^o^ has the strongest modulation. This is because the magnetization is in a canted geometry as a consequence of the noncollinear alignment of the magnetic anisotropy fields and applied field when the latter is along *θ*_H_ = 120^o^, and the strongest magnetic torque resulting in the largest precession amplitude is generated when the anisotropy fields undergo the strongest modulation. As mentioned above in the Section 2.3, the uniaxial magnetic anisotropy is present in Co/BiFeO_3_ owing to the strain along the [010] axis caused by the spontaneous FE polarization. From Equation 4, this FE polarization with its resultant strain has the largest modulation for *β*_E_=0^o^ or 90^o^. As a consequence, the uniaxial magnetic anisotropy may be modulated at the largest scale and the precession amplitude presents extrema at these *β*_E_ values, as shown in Figure 3b. Therefore, the sinusoidal variations of the precession amplitude and the FE polarization induced *θ*_K_ peak with *β*_E_ exhibit 45^o^ phase shift.

Although the modulated FE polarization plays an important role in generating the strain‐related transient anisotropy fields to trigger the magnetization precessions, it greatly attenuates within a few picoseconds as shown in Figure 4a. At the longtime scale over 100 ps, the strain‐modulated anisotropy field is negligible and thus no apparent difference of the precession frequency is observed for various *β*_E_. Actually, it was found in BiFeO_3_ film that the ORE‐induced strain affects the coherent acoustic phonons mainly within 50 ps.^[^ ^36^ ^]^ Nevertheless, we still observe a small long‐lived dc signal with the sinusoidal variation with *β*_E_, presenting opposite phase with that of the peak *θ*_K_ signal, as shown in Figures 3c and 4b. This long‐lived dc signal is likely due to the residual strain and the converted heat effect from the transient FE polarization, producing an extra bifringence effect and thus resulting in the opposite sign of *θ*_K_ as compared to that induced by the transient FE polarization. Furthermore, based on the overall shape and phase correlation between the *β*_E_ dependences of precession relaxation time *τ* and the transient FE polarization and long‐lived dc signals, we infer that the damping of magnetization precession is affected by the residual strain proportional to the transient FE polarization.

The nonthermal modulation of the magnetization precession presented in our case is ≈20% of the precession excitation independent on the laser polarization, as can be seen from Figure 3b. The latter is contributed from the heat‐induced demagnetization and magnetic anisotropy modification.^[^ ^26^, ^40^ ^]^ We believe that the relatively smaller nonthermal origin stems from the weak magnetic anisotropy relevant to the FE polarization as a result of the nonperfect single crystalline structure of the Co film and the existence of multidomain FE state in BiFeO_3_. We anticipate that the nonthermal modulation of the magnetization dynamics can be greatly enlarged in samples with the improved crystallinity and single domain structure of large in‐plane FE polarization. Actually, the helicity‐dependent magnetization modulation is much weaker compared to the heat‐driven demagnetization in the pure metallic ferromagnet under laser excitation. The deterministic magnetization reversal due to the inverse Faraday effect reported in reference [22] relies on the ultrafast heating of the medium close to the Curie temperature. Moreover, for the pure metallic ferromagnet, the helicity‐dependent magnetization precession amplitude is typically much smaller than that of the precession driven by the heating effect, as illustrated in Figure S2 (Supporting Information), and thus it is usually overwhelmed by the latter and can be hardly observed. It is thus highly worthy of exploration of the ultrafast magnetization control by the linearly polarized light in the optimized FM‐FE coupled heterojunctions in the future.

As mentioned in the introduction, it was reported in reference [25] that the light can induce the strain in the Ni/BiFeO_3_ bilayer and thus lead to the modulation of magnetic properties, however, its magnetic response was found to occur at a quite long‐timescale of second because the photo‐striction effect was due to the photocurrent across the BiFeO_3_ crystal, which may take quite a long time to accumulate the carriers, build the electric field, and then influence the net polarization in BiFeO_3_ to yield strain. This process does not rely on the pump light polarization. In contrast, our work shows a very fast magnetic response at the time scale of picosecond and it has a pronounced dependence on the light polarization direction. The underlying mechanism is due to the optical rectification effect with the intrinsic ultrafast response. We believe the ultrafast response provides a perspective for light‐controlled magnetic recording and spintronic devices on sub‐ns timescale.

## 3 Conclusion

In summary, we have demonstrated that the optically driven magnetization precession in the FM Co coupled with the FE BiFeO_3_ is correlated to the linear polarization angle (*β*_E_) of the pump pulses. The precession amplitude shows the sinusoidal dependence on *β*_E_ with the period of 180^o^. By comparing the polarization‐dependent ultrafast dynamics of the pure FE BiFeO_3_ film, it is shown that this ultrafast nonthermal control of the magnetization precession excitation in the FM‐FE coupling system originates from the ORE‐induced FE polarization of the BiFeO_3_ layer. The transient FE polarization depends on the pump light polarization and the subsequent electrostriction effect induces the corresponding strain in the bilayer. This strain modulates the magnetic anisotropy of the Co layer as the sinusoidal function of *β*_E_, leading to the sinusoidal modulation of the magnetization precession amplitude with *β*_E_. Our work opens a new avenue to manipulate ultrafast magnetization dynamics using the linearly polarized light and should advance studies of nonthermal control of ultrafast magnetization dynamics for magnetic storage technology.

## 4 Experimental Section

### Sample Preparation

To prepare the Co/BiFeO_3_ bilayer sample, the pulsed laser deposition was used with the laser pulse wavelength of 248 nm and energy of 290 mJ to grow a FE BiFeO_3_ layer with a thickness of 80 nm on Nb‐doped (001) oriented single crystal STO substrate. Before the growth, the substrate was heated to 700 °C, and the BiFeO_3_ was then deposited at a pressure of 2.0 Pa at a growth rate of 6 nm min^−1^. After annealing at a pressure of 9 × 10^−4^ Pa for 30 min, the substrate was naturally cooled to room temperature. Then the sample was transferred to the magnetron cavity, and the FM Co layer with a thickness of 4 nm was sputtered by magnetron sputtering at room temperature with a growth pressure of 4 × 10^−4^ Pa. During the growth of the Co layer, an external field of 200 Oe was applied along the [110] axis of BiFeO_3_. The metal Ta was chosen as the top protective layer with a thickness of 1.5 nm.

### Time‐Resolved MOKE Measurements

In the TRMOKE system, a Ti: Sapphire laser amplifier delivers laser pulses with the pulse width of ≈100 fs, the laser repetition rate of 1 kHz, and central wavelength of 800 nm. The 400 nm laser pulses, used as the pump light incident perpendicularly on the sample, were generated by frequency doubling the 800 nm pulses in a beta barium borate (BBO) crystal. Before incidence on the sample, the pump laser transmitted a polarizer followed by a zero‐order half‐wave plate to tune its polarization angle (*β*_E_). The pump fluence on the sample was kept constant at ≈0.5 mJ cm^−2^. The probe laser pulses of 800 nm were incident at a 45° oblique angle, and the transient polarization rotation (*θ*_K_) of the reflected probe pulses was detected by a balanced detector in the combination of a half‐wave retarder and a Wollaston prism. In the TRMOKE measurements, a vector magnetic field was applied within the sample plane along a certain direction (*θ*_H_) to align the magnetization of Co to the desired orientation (*θ*_M_).

## Conflict of Interest

The authors declare no conflict of interest.

## Supporting information

Supporting Information

## Data Availability

The data that support the findings of this study are available from the corresponding author upon reasonable request.
