# Supplementary material for: Nonthermal Ultrafast Optical Control of Magnetization Dynamics by Linearly Polarized Light in Metallic Ferromagnet
Source: Adv Sci (Weinh). 2023 Jan 3;10(6):2205903. doi: 10.1002/advs.202205903 (PMC9951311; doi:10.1002/advs.202205903)
Supplement: Supplementary file 1 — Supporting Information [file ADVS-10-2205903-s001.pdf]

Supporting Information

Title

Nonthermal ultrafast optical control of magnetization dynamics by linearly polarized light in metallic ferromagnet

Authors

Jingyu Shi†, Zirui Zhao†, Yu Dai, Jiang He, Tao Li, En Liang, Jun Wang, Gang Ni*, Chuanxiang Sheng, Di Wu, Shiming Zhou, Liangyao Chen, and Haibin Zhao[5]**

*Corresponding author. Email: hbzhao@fudan.edu.cn, wangjunfd@fudan.edu.cn, gni@fudan.edu.cn

†J. Shi and Z. Zhao contributed equally to this work.

Sample crystal structure characterization

The X-ray diffraction (XRD) was used to determine the diffraction pattern of $BiFeO_3$. According to the XRD peak positions of $BiFeO_3$ (001), (002), and (003) shown in Supplementary **Figure S1(a)**, the $BiFeO_3$ has the tetragonal-like single crystalline structure. In addition, the φ scanning mode of XRD (Supplementary **Figure S1(b)**) establishes that the angle between the lattice principle axis and the actual boundary of the sample is 0°, which means that the principle axis of the $BiFeO_3$ lattice and the actual boundary of the sample completely coincide. In addition, it can be seen that the φ scanning peak of the single crystal substrate $SrTiO_3$ corresponds exactly to the peak position of $BiFeO_3$, indicating that the two sets of lattice axes coincide and match perfectly.

Optical Kerr effect

We found in Figure 5(b) of the main article that there are two θ_K peaks defined as peak I at t≈0.5 ps and peak II at t≈0.2 ps. Both peaks display a pronounced sinusoidal dependence on β_E with the period of 180°. The peak II emerges within the laser pulse interaction duration, pointing to the optical Kerr effect. For the optical Kerr effect, the polarization rotation angle η of the probe light induced by the linearly polarized pump light conforms to the following formula [Refs. 33, 34 in the main text]

$$\eta = -\frac{32\pi^2 I_{pump}}{c|1+n|^2}\chi^* \times \sin 2\delta \quad \text{(S1)}$$

where I_{pump} is the intensity of the pump laser pulse, c is the speed of light, n is the complex refractive index of the material, χ^* is the effective third-order susceptibility coefficient, and δ is the angle between the directions of pump and probe light polarizations. In our experiments, the incident probe laser is *p*-polarized, and thus δ is equal to 90° for *s*-polarized pump laser with $\beta_E = 0^o$. For the change of β_E from 0° to 360°, the variation profile of η is fully consistent with the experimental results shown in Figure 5(d) of the main article. Therefore, we believe that the peak II in the ultrafast process of $BiFeO_3$ stems from the optical Kerr effect.

Supporting Display items

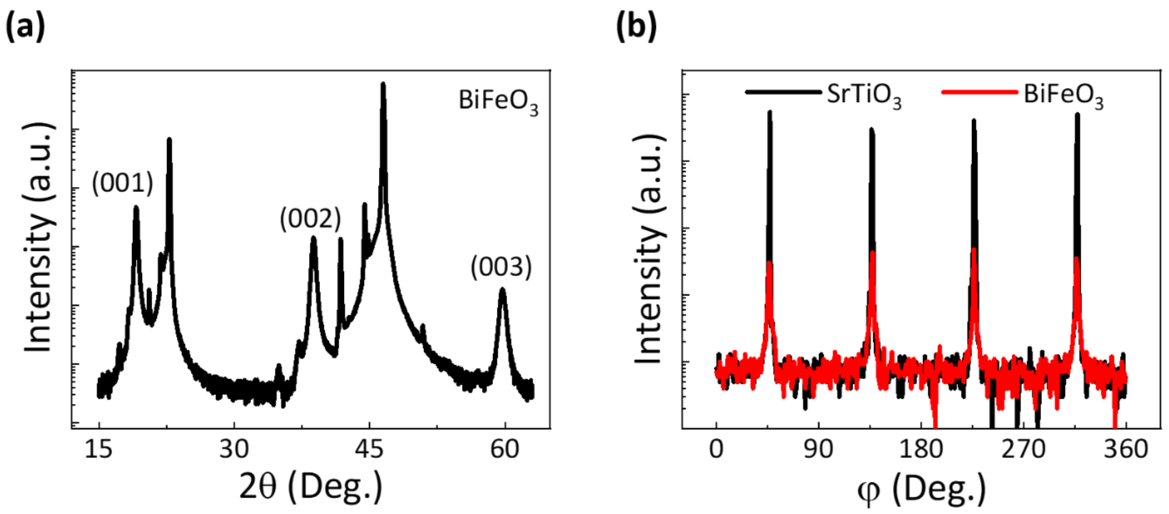

Figure. S1. a) XRD patterns for $BiFeO_3$ film. b) XRD φ-scan for $BiFeO_3$ film and $SrTiO_3$ substrate.

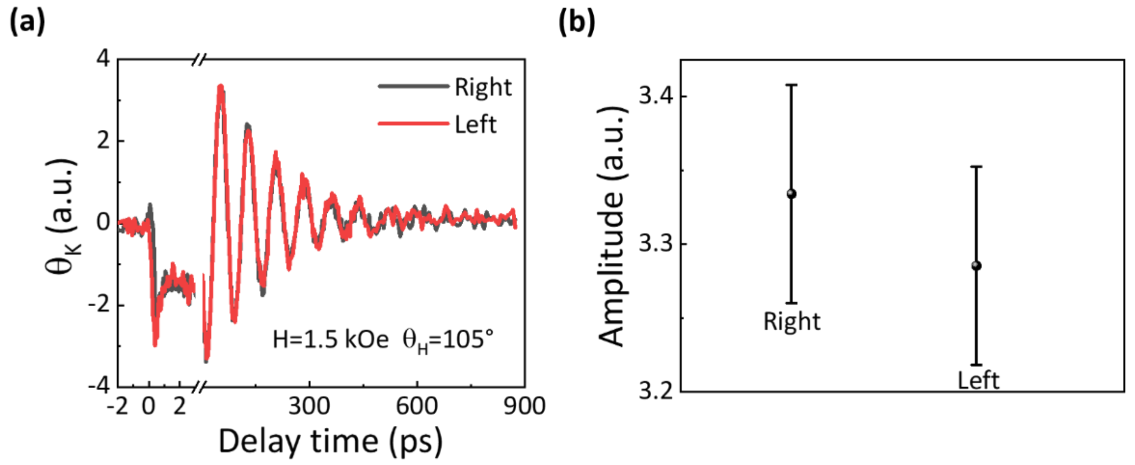

Figure S2. Pump helicity dependence of the ultrafast magnetization precession dynamics in Co/$BiFeO_3$. **a**) θ_K(t) in Co/$BiFeO_3$ at H=1.5 kOe and θ_H=105°. Red curve: LCP pumping. Black curve: RCP pumping. **b**) The precession amplitude of LCP and RCP pumping.

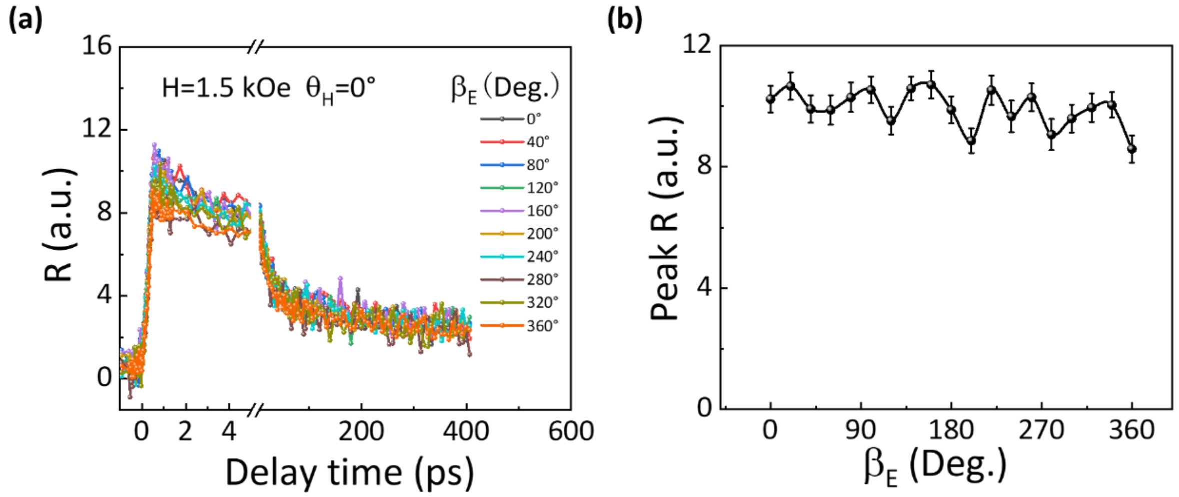

Figure S3. Pump polarization dependence of transient reflectivity in $BiFeO_3$. **a**) θ_K(t) in $BiFeO_3$ for $t \leq 400$ ps. **b**) β_E-dependence of peak θ_K values at t=0.5 ps obtained from (a).
